# Optimization method for determining vertices in lattice radiotherapy

**DOI:** 10.3389/fonc.2025.1582402

**Published:** 2025-04-30

**Authors:** Pan Ma, Yingjie Xu, Yuhe Yao, Ningning Lu, Jianrong Dai

**Affiliations:** Department of Radiation Oncology, National Cancer Center/National Clinical Research Center for Cancer/Cancer Hospital, Chinese Academy of Medical Sciences and Peking Union Medical College, Beijing, China

**Keywords:** optimization, adaptive simulated annealing, closest packing, lattice, peak-to-valley index

## Abstract

**Purpose:**

This study presents an optimization method for arranging lattice radiotherapy (LRT) targets to enhance the contrast between peak and valley doses, aiming to improve the treatment effectiveness and precision.

**Materials and methods:**

The LRT target comprises multiple sphere-like vertices generated using the optimization method, which involves four steps: 1) generating a volume for vertex arrangement, 2) determining initial positions and size of packing units, 3) determining initial positions and size of all the vertices and 4) optimizing the final vertex positions by using adaptive simulated annealing (ASA). Volumetric modulated arc therapy plans were retrospectively regenerated using the initial vertices produced by closest packing (Plan_Clo) and vertices obtained after ASA optimization (Plan_Opt). The peak-to-valley index (PVI) that characterizes the difference between peak and valley doses was introduced to evaluate the performance.

**Results:**

A statistically significant difference was observed in the average PVI between Plan_Clo and Plan_Opt (p = 0.000). The average PVI ratio for Plan_Opt compared to Plan_Clo was 5.95 ± 4.87 (range: 1.24–16.80).

**Conclusion:**

The proposed optimization method for determining LRT target vertices has been validated, demonstrating a significant improvement in the PVI. ASA optimization, combined with closest packing, effectively enhanced the peak-to-valley dose difference in LRT, showcasing its potential for advancing treatment planning.

## 1 Introduction

Spatially fractionated radiotherapy (SFRT) involves delivering highly nonuniform tumor dose distributions, substantially differing from regular radiotherapy that seeks the highest possible dose homogeneity (1–3). SFRT was first demonstrated in 1909 by Alban Köhler, a pioneering German radiologist who developed grid radiotherapy (4). Over more than a century of development, four main types of SFRT techniques have emerged: grid (5–7), lattice (2, 8–10), microbeam (11) and minibeam (12). The grid and lattice techniques are clinically used, particularly for treating bulky tumors, showing significant and sometimes drastic palliative tumor responses with minimal toxicity (13–21). Grid radiotherapy using multi-leaf collimators is more widely available and offers improved dosimetry compared with radiotherapy using physical grid blocks. However, two-dimensional grid radiotherapy remains challenging, as normal tissues are exposed to high radiation doses, with the highest doses being delivered to superficial tissues outside the target volume.

Introduced as a modern 3D SFRT technique, lattice radiotherapy (LRT) offers flexibility to achieve the intended SFRT goals by generating desired nonuniform dose distributions using an inverse planning approach. LRT planning defines high dose vertices as inverse optimization targets consisting of sphere-like sub-volumes (i.e., vertices) with diameters of approximately 1 cm and separation between dose vertex centers of approximately 2–5 cm (2, 14, 22).

In LRT, no rigorous requirements are imposed for either the symmetry of placing high dose vertices or the uniformity of their size and shapes. Instead, the number of high dose vertices depends on the size and shape of the tumor volume as well as the resolution of beam apertures.

The prescription of an LRT plan requires specifying the peak, valley, and tumor peripheral (i.e., normal tissue) doses. The peak dose is prescribed to cover 95% of the high dose vertices, and the normal tissue dose specifies the maximum allowable dose around the tumor margin to control toxicity. While meeting the prescription requirements of the high dose vertex and normal tissue doses, planning is aimed at minimizing the valley dose and increasing the difference between the peak and valley doses.

Currently, oncologists arrange vertices manually using simple geometric tools, such as distance measurement, available in treatment planning systems (TPSs) (14, 22). This kind manual method is time-consuming, error-prone, inaccurate, and poorly reproducible. In addition, it hinders data traceability and auditing, and it fails to suitably handle complexity. During the placement of vertices, several key issues need to be addressed, such as determining the initial vertex positions inside the gross tumor volume (GTV), optimizing the angle for arranging a layer of vertices on the axial plane, and selecting the appropriate diameter and spacing of vertices.

Tucker et al. (23) employed a script to automatically generate SFRT spheres by rotating the vertices of the lattice grid about the craniocaudal axis in 10° increments up to 90° and then translating in 2–3 mm increments along 3 cm on the axial planes of a computed tomography (CT) scan, notably enhancing the positioning efficiency. Similarly, Zhang et al. (24, 25) optimized vertex positions considering the peak-to-valley dose and organ at risk sparing as optimization objectives.

The closest packing of equally sized spheres in three-dimensional space is achieved through face-centered cubic (FCC) or hexagonal close packing (HCP) arrangements, both occupying approximately 74% of the space (26). In these configurations, each sphere is surrounded by 12 neighboring spheres, resulting in a coordination number of 12. Duriseti et al. (14)applied this method to the target volume delineation in SFRT.

In this study, we adopted the closest packing method along with an optimization method to automatically determine vertices in LRT, aiming to increase the difference between the peak and valley doses.

## 2 Materials and methods

The considered LRT target comprises multiple sphere-like vertices generated using HCP closest packing and an optimization method. Closest packing involves three steps: 1) contracting the GTV inward to generate the volume for vertex arrangement, 2) determining the initial positions and size of packing units (PUs), and 3) determining initial positions and size of all the vertices. Next, the optimization method adds a step of 4) optimizing the final vertex positions.

### 2.1 Volume for vertex arrangement

To obtain radiotherapy data, medical imaging techniques such as CT, magnetic resonance imaging, ultrasound, and positron emission tomography/CT are commonly used to create images of normal tissues and tumors. By analyzing the relative positions of normal tissues and tumors, the volume for placing LRT vertices (VPV) can be generated. As SFRT delivers a high dose, the arrangement of high dose LRT vertices should remain sufficiently distant from normal tissues to ensure protection. The corresponding distance is related to the GTV, and the boundary is determined by contracting the GTV inward by margin $m_{inward}$ derived from fitting data in Ref. (2) as follows (Equation 1):


(1) $$m_{inward}\;=\{\begin{array}{c} \;1,\;\\ \;0.00125V+0.75,\\ \;2,\; \end{array}\;\begin{array}{c} \;V<200,\;\\ 200\leq V<1000,\\ \;V\geq 1000, \end{array}$$

where *V* is the GTV in cubic centimeters.

### 2.2 Initial position and size of vertices

#### 2.2.1 Closest packing

We adopt HCP packing to arrange the sphere-like vertices for LRT planning. The sphere arrangement can be visualized as a stack of close-packed layers, with each layer containing spheres arranged in a hexagonal pattern. The layers are arranged such that the spheres in one layer fit perfectly into the spaces between the spheres in the surrounding layers, resulting in a very efficient packing. The layers in HCP stacking are arranged as ABAB…, where A layers contain spheres at the corners and B layers contain vertices at the face centers. Then, the sequence of A and B layers repeats.

**Figure 1:**
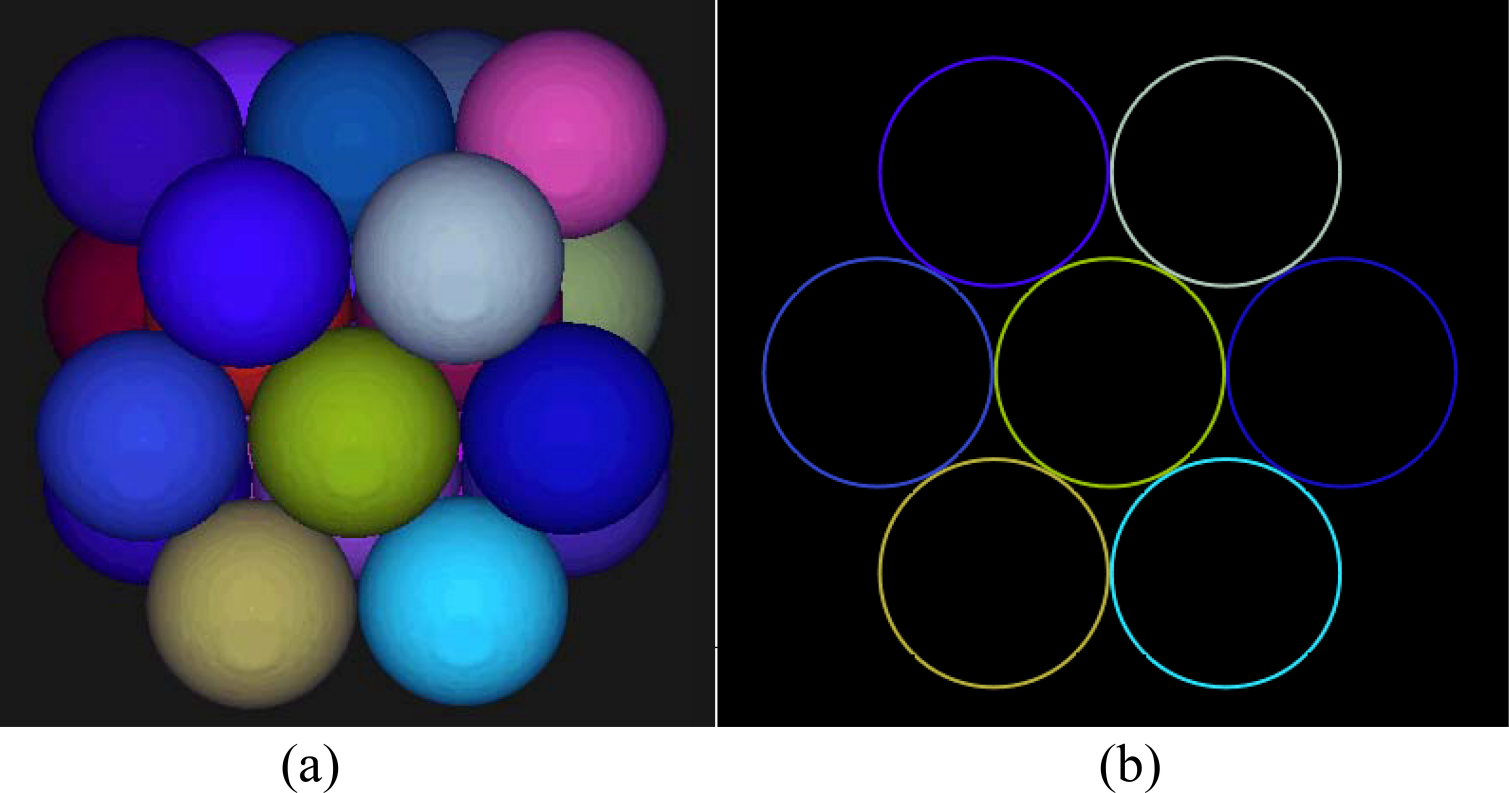
The three-layer spheres arranged using the HCP packing method **(a)** and the sphere contour drawn through the plane of the center of the outermost layer **(b)**.

The three-layer spheres, i.e., the PUs, are arranged using HCP packing as illustrated in **Figure 1a**. The sphere contours through the plane of the center of the outermost layer are shown in **Figure 1b**, with the middle circle being tangent to the surrounding six circles and indicating closest packing. In closest packing, each sphere is surrounded by up to 12 spheres.

#### 2.2.2 Initial position and size of vertices

To determine the centers of all the PUs, closest packing involves two steps: 1) determining the center of the first PU and 2) stacking the remaining PUs around the first PU. For convenience, the first PU center is located at the geometric center, ( $x_{0}$ , $\;y_{0}$ , $\;z_{0}$ ), of the VPV. The PU diameter d in centimeters is calculated using Equation 2 and derived from fitting data in Ref. (2):


(2) $$\;d=\{\begin{array}{c} \;2,\;\\ \;0.002V+1.9,\\ \;4,\; \end{array}\;\begin{array}{c} \;V<50,\;\\ 50\leq V<1000,\\ \;V\geq 1000. \end{array}\;$$

The number of rows, columns, and layers of the remaining PUs are related to the VPV, (*x*, *y*, *z*), and the center of the first sphere, ( $x_{0}$ , $\;y_{0}$ , $\;z_{0}$ ), as follows (Equations 3–8):


(3) $$LineNum_{1}=\lceil \frac{\left|\max\left(x\right)-x_{0}\right|}{\frac{\sqrt{3}}{2}d}\rceil +2,LineNum_{2}=\lceil \frac{|\min\left(x\right)-x_{0}|}{\frac{\sqrt{3}}{2}d}\rceil +2,$$

(4) $$RowNum_{1}=\lceil \frac{\left|\max\left(y\right)-y_{0}\right|}{d}\rceil +2,\;RowNum_{2}=\lceil \frac{\left|\min\left(y\right)-y_{0}\right|}{d}\rceil +2,$$

(5) $$LayerNum_{1}=\lceil \frac{\left|\max\left(z\right)-z_{0}\right|}{\frac{\sqrt{3}}{2}d}\rceil +2,LayerNum_{2}=\lceil \frac{\left|\min\left(z\right)-z_{0}\right|}{\frac{\sqrt{3}}{2}d}\rceil +2,$$

where *Line* $Num_{1}$ , *Line* $Num_{2}$ , *Row* $Num_{1}$ , *Row* $Num_{2}$ , *Layer* $Num_{1}$ , and *Layer* $Num_{2}$ are the number of columns, rows, and layers in the left, right, anterior, posterior, superior, and inferior directions, respectively, expressed a coordinate system with respect to the patient. The PUs have spatial repetition every two layers, and 2 is used to ensure a complete search space for subsequent optimization.

The coordinates of the center, $(x_{i}$ , $\;y_{i}$ , $\;z_{i})$ , of packed sphere *i* can be calculated as follows:


(6) $$x_{i}=\{\begin{array}{c} x_{0}+\left(i-1\right)d,\;1\leq i\leq LineNum_{1}\\ x_{0}+\left(i+1\right)d,\;-1\leq i\leq -LineNum_{2} \end{array},$$

(7) $$y_{i}=\{\begin{array}{c} y_{0}+\left(i-1\right)d,\;1\leq i\leq RowNum_{1}\\ y_{0}+\left(i+1\right)d,\;-1\leq i\leq -RowNum_{2} \end{array},$$

(8) $$z_{i}=\{\begin{array}{c} z_{0}+\left(i-1\right)d,\;1\leq i\leq LayerNum_{1}\\ z_{0}+\left(i+1\right)d,\;-1\leq i\leq -LayerNum_{2} \end{array}.$$

**Figure 2:**
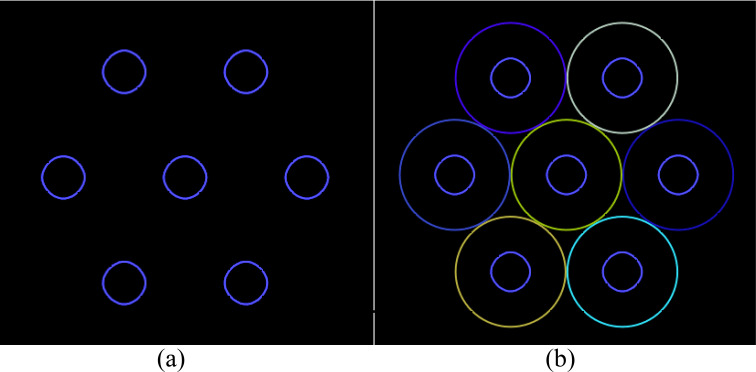
The initial arrangement of the vertices **(a)** generated by placing a vertex at the center of each PU **(b)**.

LRT planning involves sphere-like vertices similar to the PUs. Hence, the sphere centers obtained by closest packing are the sphere-like vertex centers in the positions of the vertices, as shown in **Figure 2**.

The size of the vertices is determined by the size of the tumor, whose diameter is calculated based on data fitting in Ref. (2) as follows:


(9) $$d^{\prime }=\{\begin{array}{c} \;0.5,\;\;V<50,\\ 0.001V+0.4258,\;50\leq V<1000,\\ \;1.5,\;\;V\geq 1000, \end{array}$$

where $d^{\prime }\;$ is the diameter of the sphere-like vertices in centimeters. The vertex layer is parallel or perpendicular to the transverse plane of the patient image. If the total volume of vertices calculated within the VPV using Equation 9 is less than *V*_0_ (*V*_0_ is 1% of the GTV in this study), $d^{\prime }$ can be incrementally adjusted, for example, by 1 mm, until the total vertex volume reaches *V*_0_. This method determines the initial vertex diameter.

### 2.3 Final positions of vertices

#### 2.3.1 Adaptive simulated annealing

For optimization, adaptive simulated annealing (ASA), a variant of conventional simulated annealing, is adopted owing to its three key advantages: 1) different parameters can employ distinct cooling schedules, enabling faster annealing compared with Boltzmann annealing; 2) a reannealing feature enables adaptive sensitivity changes in a multidimensional solution space; 3) over 100 options are available for tuning various classes of nonlinear stochastic systems (27). These advantages, along with a publicly available source code (http://www.ingber.com), make ASA applicable to numerous scientific fields, including radiotherapy.

We apply ASA to optimize the vertex positions for LRT planning. This method adjusts the initial positions of the vertices by translating and rotating the vertices while maintaining a constant total volume. The optimization objective is increasing the distance between vertices to maximize the difference between peak and valley doses.

The distance to be optimized is expressed as (Equation 10)


(10) $$\max\;d(x^{\prime },y^{\prime },z^{\prime },\alpha ,\beta ,\gamma ),$$

where ( $x^{\prime },y^{\prime },z^{\prime }$ ) represents the positions of the first PU and (*α*, *β*, *γ*) represent the angles of the closest packing layer.

The closest packing arrangement exhibits repeatability in its pattern, ensuring that the vertices can be rotated and translated within a certain range to achieve the optimal solution. The following constraints are considered (Equations 11–13):


(11) $$(x_{i},\;y_{i},\;z_{i})\in \text{VPV},$$

(12) −180°≤α, β, γ∈≤180°,

(13) $$V=0.01V_{GTV},$$

where ( $x_{i},\;y_{i},\;z_{i}$ ) represents the coordinates of the points within vertex *i*, and $V\text{ and }V_{GTV}$ are the total volume of all the vertices and GTV, respectively.

#### 2.3.2 Optimization procedure

**Figure 3:**
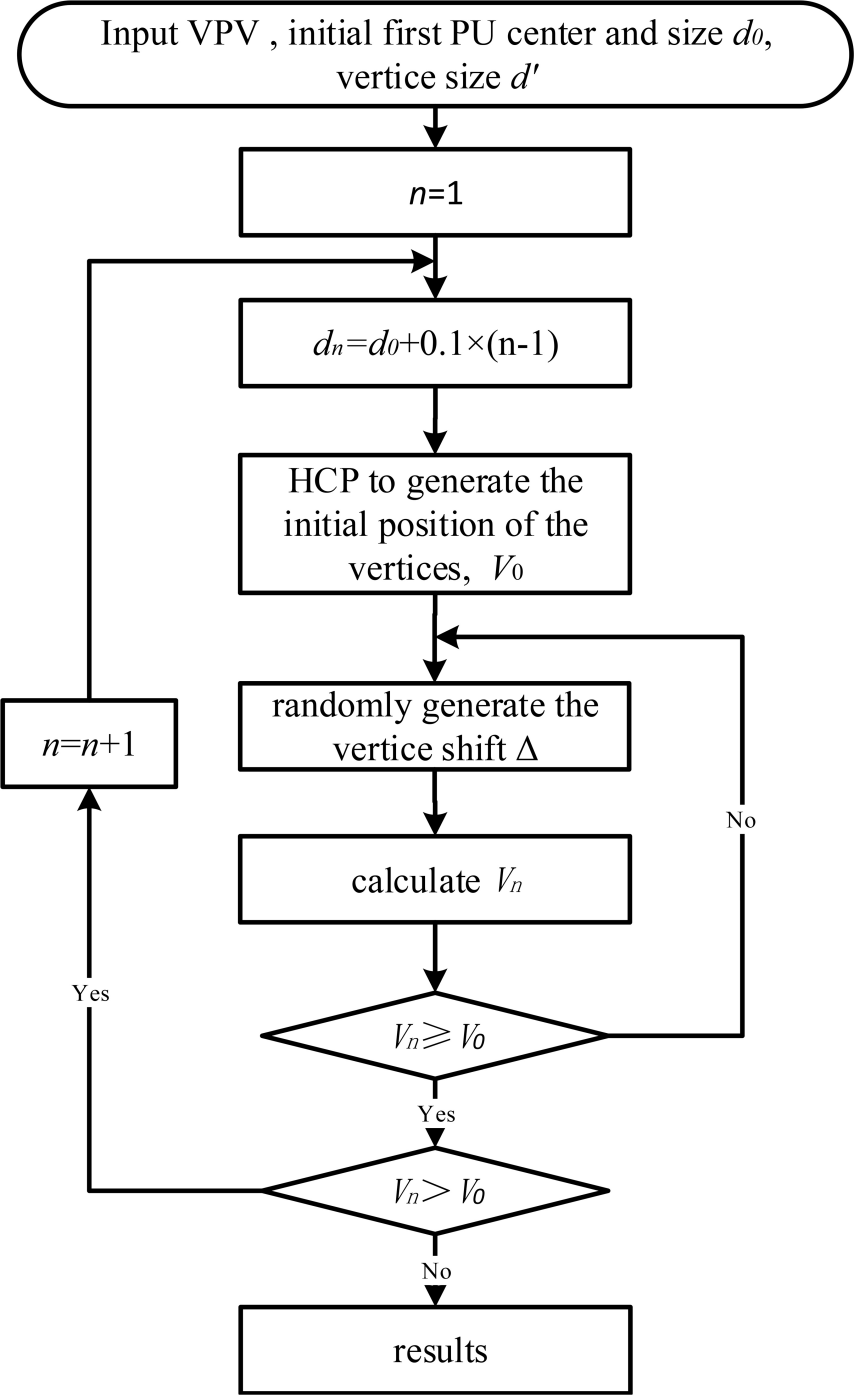
The flow chart of the adaptive simulated annealing optimization method.

ASA optimization comprises the following eight steps (**Figure 3**):

1. Input data of VPV, center position ( $x_{0}$ , $\;y_{0}$ , $\;z_{0}$ ), initial diameter $d_{0}$ of first PU, and diameter *d’* of vertices;
2. Set *n* to 1;
3. Calculate diameter of PU for step *n* as $d_{n}=d_{0}+0.1\left(n-1\right);$

4. Apply HCP closest packing to generate initial positions of vertices and calculate initial volume $V_{0}$ ;
5. Using ASA, adjust the temperature parameters to control the probability distribution of random numbers within the domain defined by Δx∈ (- $\sqrt{3}d_{n},\;\sqrt{3}d_{n}$ ), Δy∈ (- $d_{n},\;d_{n}$ ), Δz∈ (- $\sqrt{3}d_{n},\;\sqrt{3}d_{n}$ ), Δα∈ (- π, π ), Δβ∈ (- π, π ), and Δγ∈ (- π, π ). Randomly generate displacement ( Δx,Δy,Δz,Δα,Δβ,Δγ ) of vertex ( $x_{i}$ , $\;y_{i}$ , $\;z_{i}$ );
6. Calculate optimal target volume $V_{n}$ ;
7. If $V_{n}$ is greater than or equal to $V_{0}$ , go to step 8. Otherwise, go to step 5;
8. If $V_{n}$ is greater than $V_{0}$ , set *n* to *n* + 1 and go to step 3. Otherwise, return the position of vertices ( $x_{i}$ , $\;y_{i}$ , $\;z_{i}$ ) and terminate.

If a portion of a sphere-like vertex lies outside the VPV, the volume of that portion is subtracted, resulting in vertices that are not entirely spherical. Any volume less than 0.065 cm^3^, which is equivalent to a sphere with a diameter less than 0.5 cm, is discarded to avoid generating excessively small subfields that cannot be implemented in practice.

### 2.4 LRT planning

In this study, we considered 17 patients for retrospective LRT replanning using the Pinnacle3 TPS (version 9.16; Philips Healthcare, Andover, MA, USA), which was commissioned according to the TG-119 recommendations. The GTV and critical structures were contoured and reviewed during a chart round by radiation oncologists. For each patient, volumetric modulated arc therapy plans were designed using the initial vertices obtained from closest packing (Plan_Clo) and the final vertices after ASA optimization (Plan_Opt).

The total volume of the vertices was 1% of the GTV, and *d’* was defined accordingly.

The flattening filter-free photon beam energy for all plans was set to 6 MV for delivery using a Varian Edge linear accelerator (Varian Medical Systems, Palo Alto, CA, USA) equipped with 80 pairs of leaves, with leaf widths of 2.5 mm within the central 10 cm range and 5 mm for the remaining leaves. The dose grid resolution was 0.2 × 0.2 × 0.2 cm. Volumetric modulated arc therapy plans were generated using from three to six coplanar partial arcs (28). The delivery time was not limited. The continuous gantry motion, dose-rate variation, and multileaf collimator motion were approximated by optimizing individual beams at 3°–4° gantry angle increments.

The same dose–volume constraints were applied to all the plans during inverse planning optimization. The final dose distributions were calculated using adaptive convolution. Planning was intended to deliver a prescribed dose of 15 Gy to at least 95% of the vertices in one fraction, and the dose uniformity requirement ranged from −5% to 30%. Rings 1, 2, and 3 at distances of 5, 10, and 15 mm from the vertices were respectively generated to enhance the dose gradient and minimize the penumbra region. Additional spheres between the vertices were created to reduce the dose in the valley region. Optimization for a specific patient aimed to minimize the dose to the normal tissue outside the GTV while maintaining the maximum peak dose and minimum valley dose.

### 2.5 Evaluation measure

The valley dose, not the peak dose, has been closely associated with increased survival when compared with controls (9, 29). Although white papers are currently being defined for both grid therapy and LRT, additional advanced measures of the dose heterogeneity are needed (2, 3, 30).

The contrast between peak and valley doses is a key in SFRT planning. However, existing definitions vary: ① $D_{95}^{valley}$ (31) defines the dose covering 95% of the valley region, derived from the GTV minus the vertices with a non-uniform margin, which approximates the minimum dose of the valley region; ②VPDR_90/10_ (32)uses the D_10%_/D_90%_ thresholds, but it trends to underestimate the true peak doses; ③VPDR_Median_ (33) calculates the dose ratios between adjacent vertices’ D1% peaks and their corresponding midpoint valleys, offering a more accurate representation of spatial configurations. The absence of standardized metrics impedes inter-study comparisons and clinical optimization of therapeutic ratios between tumor control and normal tissue sparing. The absence of standardized metrics hinders meaningful comparisons across studies.

**Figure 4:**
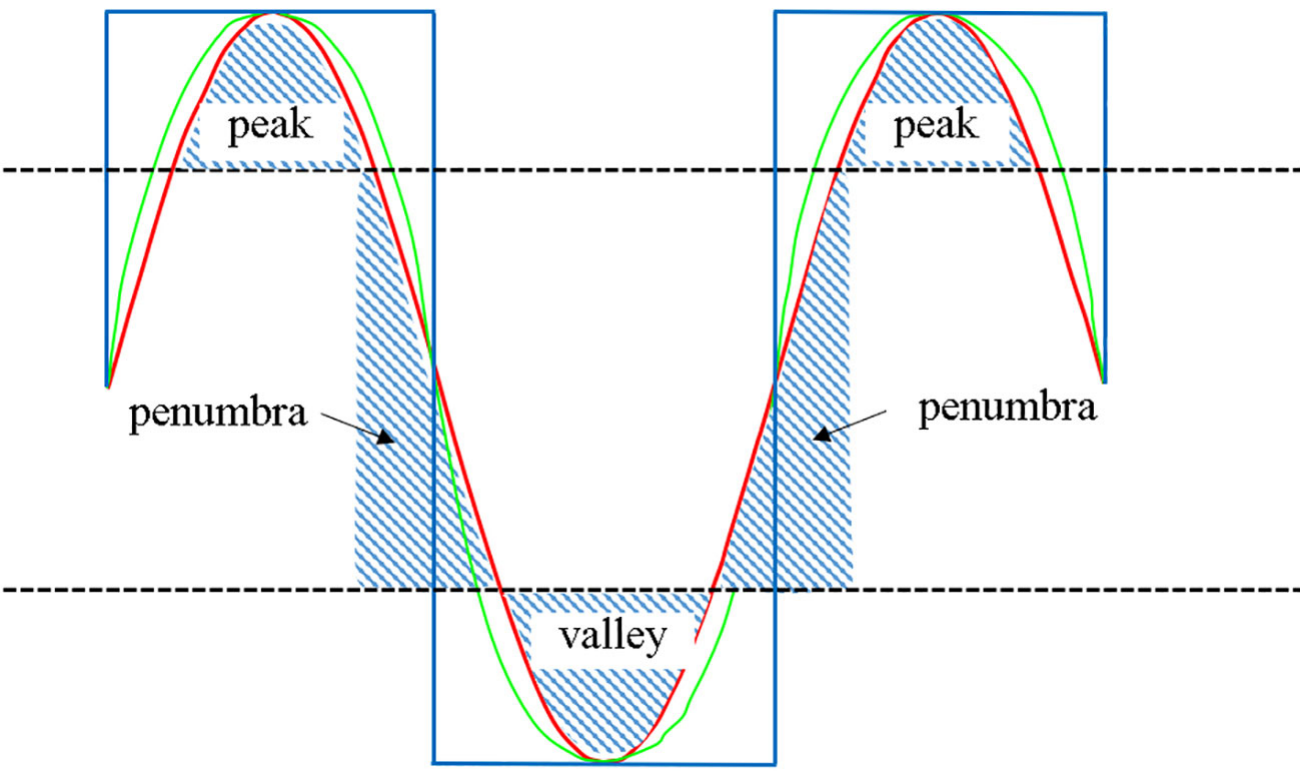
Diagram illustrating definitions of peak region, valley region, and penumbra region.

As illustrated in the **Figure 4**, the maximum and minimum doses in the red and green profile curves are identical despite the varying dose gradients. Notably, the valley region is the distinctive feature between various SFRT techniques, with a larger volume indicating a greater ability to widen the dose gap between peaks and valleys.

To precisely characterize the dose peaks and valleys, we conducted a quantitative evaluation of the dose distributions. Defining regions above 80% as peaks, those below 20% as valleys, and those between 80% and 20% as penumbra regions in the dose profile between the centers of adjacent spheres facilitated the evaluation. Large peak and valley areas along with small penumbra areas yielded more pronounced dose peaks and valleys (blue curve in extreme scenario of **Figure 4**). Accordingly, we defined the peak-to-valley index (PVI) as


(14) $$PVI=\frac{S_{\text{peak}}S_{\text{valley}}}{S_{\text{penumbra}}},$$

where $S_{peak}$ , $S_{valley}$ , and $S_{penumbra}$ are the areas of the peak, valley, and penumbra regions, respectively. A higher PVI indicates more pronounced dose peaks and valleys.

For evaluation, every vertex and its closest neighbors formed a pair of dose peak and valley, and the PVI was calculated. If a vertex had multiple neighbors, the average PVI was considered. Each plan contained multiple vertices, and the average PVI across all the vertices in the plan was computed to evaluate the plan PVI. Owing to the relative high valley doses in the LRT plans, the peak and valley dose thresholds were set to 80% and 50%, respectively. The PVI from Plan_Opt was compared with that from Plan_Clo to evaluate the optimization effectiveness.

The mean dose to the GTV and dose to 1 cm^3^ of normal tissue (NT_1cc_) were used to evaluate the differences in the internal and external doses of the GTV. Normal tissue was determined by subtracting the GTV from the patient contour outline on the GTV plane.

The Wilcoxon matched-pairs and signed-rank tests for nonparametrically distributed data were applied to compare Plan_Clo with Plan_Opt, respectively. Statistical significance was considered for *p* < 0.05 (two-tailed). All the statistical analyses were performed using SPSS Version 13.0 (SPSS, Chicago, IL, USA).

## 3 Results

### 3.1 Representative patient

**Figure 5:**
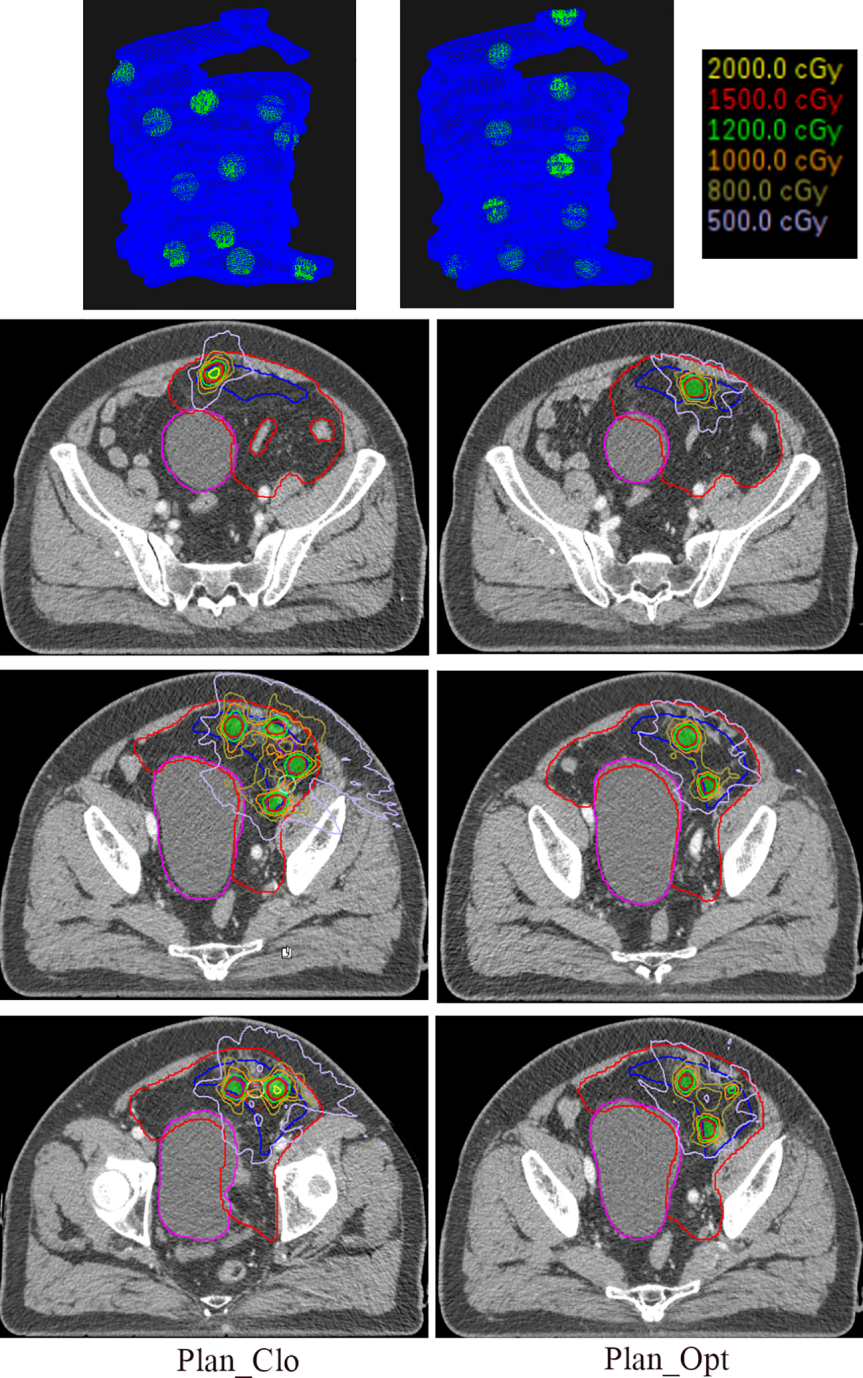
The first row of the figure illustrates the three-dimensional distribution of vertices, with the blue region indicating the VPV and the green region denoting the vertices. The remaining three rows show the isodose distributions in the central axial planes for one representative patient in Plan_Clo (left) and Plan_Opt (right). The red line represents the GTV, the blue line represents the VPV, the magenta line represents the organ at risk, bladder, and the green shaded area represents the vertices. Plan_Clo and Plan_Opt were generated using initial vertices from closest packing and optimized vertices from adaptive simulated annealing, respectively.

**Figure 5** shows the distribution of vertices in three-dimensional space (first row) and isodose distributions on the axial planes (next three rows) for a representative patient under Plan_Clo (left) and Plan_Opt (right). The red, blue, and magenta contours represent the GTV, VPV, and organ at risk (bladder), respectively, and the green shaded area represents the vertices.

The patient’s GTV was 1407.00 cm^3^, and the VPV was 320.15 cm^3^. The vertex volumes were 15.11 and 15.29 cm^3^, accounting for 1.07% and 1.09% of the GTV for 11 and 11 vertices in Plan_Clo and Plan_Opt, respectively. The vertex spacings were 36 and 41 mm for Plan_Clo and Plan_Opt, respectively. The two plans provided a vertex diameter of 15 mm.

After optimization, the PVI for Plan_Opt showed a 16-fold increase, achieving significant benefit. This result suggested that when the GTV had a more complex geometry, optimization was more effective for planning.

As shown in the three-dimensional images, the vertices for Plan_Opt were placed where the VPV shape changed considerably (e.g., vertices at the top of the images). Owing to the different vertex distribution planes in the three plans, a direct comparison of the corresponding layers was not possible; thus, only representative layers were depicted.

### 3.2 All patients

**Figure 6:**
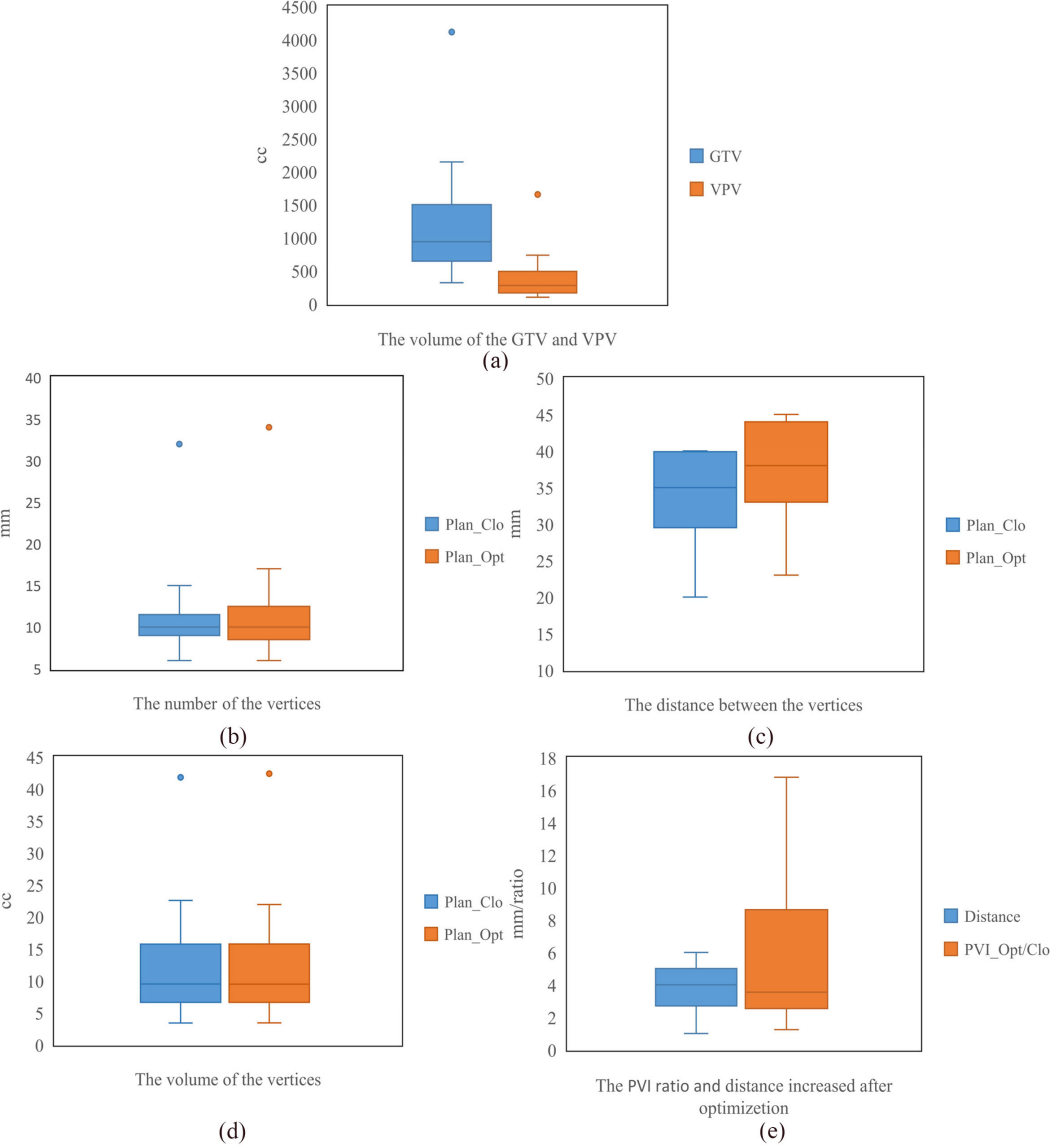
The statistics for seventeen patients, including the volumes of GTV and VPV **(a)**, the number of vertices **(b)**, the distance between vertices **(c)**, the volume of vertices **(d)**, and the *PVI* and increase in distance between vertices **(e)**. Plan_Clo and Plan_Opt were generated using initial vertices from closest packing and optimized vertices from adaptive simulated annealing, respectively.

As shown in **Figure 6**, the average GTV and VPV were 1225.46 ± 858.81 cm^3^ (range, 545.64–4113 cm^3^) and 395.33 ± 355.70 cm^3^ (range, 151.98–654.31 cm^3^). The average number of vertices per patient was 11 ± 6 (range, 7–32) for Plan_Clo and 12 ± 6 (range, 6–32) for Plan_Opt.

For Plan_Clo and Plan_Opt, the distance between vertices per patient varied, with average distances of 34.15 ± 5.79 mm (range, 20.00–40.00 mm) and 37.86 ± 6.40 mm (range, 23.00–45.00 mm) across the 17 patients, respectively.

The average volume of the vertices was 12.68 ± 8.76 cm^3^ (range, 3.4–41.81 cm^3^) for Plan_Clo and 12.66 ± 8.84 cm^3^ (range, 3.41–42.39 cm^3^) for Plan_Opt. The average total volume of the vertices showed no significant difference between Plan_Opt and Plan_Clo (*p* = 0.820). The vertex diameters were consistent across the two plans for every patient. For the 17 patients, the average diameter was 13.14 ± 2.32 mm (range, 7.5–15 mm). **Table 1** provided a clear summary of the key data extracted from **Figure 6**, making it easier for comparison and analysis.

**Table 1:** Summary of box plot statistics from **Figure 6** for GTV, VPV, vertices, and PVI optimization.

Data Category		Min Value	Q1 (First Quartile)	Median	Q3 (Third Quartile)	Max Value
Volume (cc)	GTV	319.23	658.09	938.38	1419.41	4113.00
	VPV	99.69	175.57	277.26	414.78	1654.03
Number of Vertices	Plan_Clo	6	10	11	13	31
	Plan_Opt	6	9	10	12	34
Distance Between Vertices (mm)	Plan_Clo	20	30	35	39	40
	Plan_Opt	23	34	38	44	45
Volume of the Vertices (cc)	Plan_Clo	3.4	6.96	9.49	15.26	41.81
	Plan_Opt	3.41	6.96	9.47	15.39	42.39
PVI Ratio and Distance(Ratio/mm)	PVI_Opt/Clo	1.24	2.57	3.55	6.49	16.80
	Distance	1	3	4	5	6

### 3.3 Performance of optimization method

The average increase in distance *d* between vertices was 3.7 ± 1.3 mm. The maximum increase (5 mm) was from 36 to 41 mm, and the minimum increase (1 mm) was from 40 to 41 mm.

PVI ratio was used to compare the PVI difference between Plan_Opt and Plan_Clo, for which the average PVI across the 17 patients showed a statistically significant difference (*p* = 0.000). As shown in **Figure 6e**, the average PVI ratio (PVI_Opt/Clo) between Plan_Clo and Plan_Che was 5.95 ± 4.87 (range, 1.24–16.80). After optimizing the vertex positions, the distances between the vertices increased and the PVI improved.

The average NT_1cc_ values were 27.81 and 24.88 Gy across the 17 patients for Plan_Clo and Plan_Opt, respectively, with no statistically significant difference (*p* = 0.332). The mean doses to GTV averaged 13.86 and 15.50 Gy across 17 patients for Plan_Clo and Plan_Opt, respectively, showing a statistically significant difference (*p* = 0.02).

Within the GTV, the optimized plan had a higher mean dose because vertices of the same volume were more dispersed throughout the GTV after optimization. Constraints on normal tissue were set through the objective function. Thus, the maximum dose to 1 cm^3^ of normal tissue showed no statistically significant difference between plans.

## 4 Discussion

In this study, we considered the vertices in LRT to have a sphere-like shape. If a cylinder is considered, the vertices can be automatically generated using our method, with only one plane being required to set the vertex regions. The vertex positions were also optimized while maintaining the total volume of the vertices to increase both the distance between vertices and PVI. While maintaining the total volume of the vertices, their sizes could be reduced, thereby increasing the difference between peak and valley doses.

The automatic arrangement of LRT targets allows to eliminate suboptimal arrangements by discarding individual oncologist’s preferences and ensure consistent target placement across patients, thus contributing to homogeneity across clinical trials. Manually adjusting the vertex positions is based solely on intuition, which alters the distances between the adjusted vertex and its neighboring vertices, further affecting the PVI. Introducing manual adjustments increases the influence of human factors, thereby introducing unintended plan heterogeneity. However, if a clinician determines that a particular sphere is too close to a critical organ and cannot provide adequate protection, manual adjustments may still be made. With the increasing clinical experience in applying the method proposed in this study, we aim to improve its robustness in generating vertices by refining the inward boundary adjustment during the VPV generation process, thereby minimizing the need for manual vertex adjustments.

Increasing evidence indicates that tumors exhibit large heterogeneity, leading to high variability in their dose response to radiotherapy, which can drastically impact the clinical outcomes (34–36). Generating spatially heterogeneous treatment doses that account for the dose response variability of individual tumors has clinical significance. Therefore, an alternative approach with scientific rigor and accuracy may involve positioning vertices based on metabolic data. This concept, known as metabolic-guided LRT, was explored by Ferini et al. (29). It involves targeting the locations with high 18F-FDG uptake, corresponding to areas of increased metabolic activity, to administer higher doses to the more active tumor regions. During optimization of LRT target arrangement, clinical information of this nature may be incorporated for assigning high weights to ensure that the LRT vertices are positioned in metabolically active regions.

Compared with the Tucker method (23), our approach utilizes closest packing and optimization introducing two additional rotational directions. Moreover, our method achieves higher precision in step size and rotation, providing a greater scope for finding the optimal solution. In this study, we used a computer equipped with an Intel(R) Core (TM) i7–8700 processor at 3.20 and 3.19 GHz and 24 GB of random-access memory, achieving an average optimization time of approximately 5 min. For clinical implementation, acceleration by graphics processing units may reduce the processing time.

A potential area for improving optimization is the uniformity of spacing between vertex regions. In radiotherapy, especially for techniques like SFRT that require nonuniform dose distribution, a more relaxed approach might be beneficial. In these cases, the tumor region can be partitioned into multiple sections, and within each section, vertex regions can be arranged with flexible spacing, allowing for small variations in inter-vertex distance. This relaxed approach could help achieve a better overall solution by making small compromises in certain areas, leading to larger dosimetric improvements elsewhere in the treatment volume. Such flexibility could improve the dosimetric outcomes, addressing both geometrical and clinical objectives, which are often constrained in radiotherapy optimization. Incorporating these aspects would allow for a more comprehensive treatment planning method, potentially improving treatment effectiveness by considering the underlying biological objectives, such as tumor control probability and normal tissue complication probability.

The implementation and creation of LRT fields and heterogeneous dose distributions have been facilitated by modern multileaf collimators and advanced TPSs. However, current TPSs do not fully support the creation of LRT targets or provide adequate evaluation tools for SFRT fields and plans (37). The introduction of two additional rotational directions in this study would allow a conventional TPS to assess the dose distribution only on the axial plane. Modifying the TPS to include the evaluation of dose distributions on the plane of close-packed layers as well as the PVI calculation may enable a more intuitive assessment of dose distributions in LRT planning.

## 5 Conclusion

The proposed optimization method for determining LRT target vertices has been validated, demonstrating a significant improvement in the PVI. ASA optimization, combined with closest packing, effectively enhanced the peak-to-valley dose difference in LRT, showcasing its potential for advancing treatment planning.

## Data Availability

The raw data supporting the conclusions of this article will be made available by the authors, without undue reservation.
